# Outer membrane protein complex as a carrier for malaria transmission blocking antigen Pfs230

**DOI:** 10.1038/s41541-019-0121-9

**Published:** 2019-07-08

**Authors:** Puthupparampil V. Scaria, Christopher G. Rowe, Beth B. Chen, Olga V. Muratova, Elizabeth R. Fischer, Emma K. Barnafo, Charles F. Anderson, Irfan U. Zaidi, Lynn E. Lambert, Bob J. Lucas, Debbie D. Nahas, David L. Narum, Patrick E. Duffy

**Affiliations:** 1 Laboratory of Malaria Immunology and Vaccinology, NIAID/NIH, 29 Lincoln Drive, Building 29B, Bethesda, MD 20892 USA; 2 Rocky Mountain Laboratory, NIAID/NIH, Hamilton, MT 59840 USA; 3 Merck & Co., Inc, Kenilworth, NJ USA

**Keywords:** Conjugate vaccines, Protein vaccines, Malaria

## Abstract

Malaria transmission blocking vaccines (TBV) target the mosquito stage of parasite development by passive immunization of mosquitoes feeding on a vaccinated human. Through uptake of vaccine-induced antibodies in a blood meal, mosquito infection is halted and hence transmission to another human host is blocked. Pfs230 is a gametocyte and gamete surface antigen currently under clinical evaluation as a TBV candidate. We have previously shown that chemical conjugation of poorly immunogenic TBV antigens to Exoprotein A (EPA) can enhance their immunogenicity. Here, we assessed Outer Membrane Protein Complex (OMPC), a membrane vesicle derived from *Neisseria meningitidis*, as a carrier for Pfs230. We prepared Pfs230-OMPC conjugates with varying levels of antigen load and examined immunogenicity in mice. Chemical conjugation of Pfs230 to OMPC enhanced immunogenicity and functional activity of the Pfs230 antigen, and OMPC conjugates achieved 2-fold to 20-fold higher antibody titers than Pfs230-EPA/AdjuPhos^®^ at different doses. OMPC conjugates were highly immunogenic even at low doses, indicating a dose-sparing effect. EPA conjugates induced an IgG subclass profile biased towards a Th2 response, whereas OMPC conjugates induced a strong Th1-biased immune response with high levels of IgG2, which can benefit Pfs230 antibody functional activity, which depends on complement activation. OMPC is a promising carrier for Pfs230 vaccines.

## Malaria transmission blocking vaccine: How bacteria can help

Malaria transmission blocking vaccines (TBV) target *Plasmodium* stages that transmit between human and mosquitos in order to interrupt the parasite’s life cycle and reduce spread. One TBV antigen currently under clinical development is Pf230, which is expressed on sexual *Plasmodium* stages. In this study, led by Patrick Duffy from the NIAID, researchers improve immunogenicity of Pf230. They chemically conjugate a part of Pf230 to membrane vesicles derived from bacteria, so-called outer membrane protein complexes (OMPC). Immunization of mice with Pf230-OMPC elicits a higher antibody response and a more balanced IgG subclass profile than control immunizations. Serum from Pf230-OMPC-vaccinated mice efficiently blocks infection of mosquitoes. These results with mice encourage further pre-clinical and clinical characterization of OMPC as a carrier for TBV antigens.

## Introduction

Malaria transmission blocking vaccines (TBV) are designed to interrupt the mosquito stage of the *Plasmodium* parasite life cycle; vaccinated humans pass antibodies to mosquitos during their blood meals which block parasite development in the midgut.^1–3^ By halting transmission from humans to mosquitos, TBVs could contribute to malaria elimination from affected communities and malaria eradication worldwide. Several proteins expressed during parasite sexual stages have been explored as TBV antigens.^4–17^ Among them, the zygote surface protein Pfs25 has undergone extensive preclinical studies^4,6^ and advanced to clinical testing and field trials.^18–21^ Two gametocyte/gamete surface antigens, Pfs230 and Pfs48/45, shown to be potent TBV antigens, are also leading vaccine candidates.^8,9,13–15,17^ Other TBV antigens under preclinical evaluation include the mosquito antigen AnAPN1 involved in ookinete invasion of the mosquito midgut,^10^ the zygote/ookinete surface antigen Pfs28,^7,11^ and the gametocyte surface antigen Pfs47 involved in evasion of the mosquito immune system.^5,12,16^

An effective malaria vaccine should provide a strong, durable, and functional antibody response that blocks parasite development. One of the challenges in TBV development has been achieving protein expression in correctly folded conformation, which is complex due to multiple disulfide bonds. Therefore, the actively pursued TBV antigens have been relatively small proteins (Pfs25) or smaller domains of larger proteins (Pfs230, Pfs48/45 etc.).^8,9,13–15,22,23^ These have proven to be only modestly immunogenic, presumably due to their small size and dearth of T cell epitopes. One strategy to improve the immunogenicity of poorly immunogenic antigens includes presentation in nanoparticles.^24–28^ We have shown that chemical conjugation of some TBV antigens to carrier proteins (and other macromolecules) generates nanoparticles with enhanced immunogenicity,^29–34^ and a 22 kDa recombinant protein from domain-1 of Pfs230, conjugated to EPA, is currently being evaluated in clinical trials (clinicaltrial.gov ID: NCT02334462; NCT02942277). Presentation of antigens in a particulate structure or on the particle surface allow them to be more efficiently internalized by antigen-presenting cells (APCs) than soluble antigen.^35^ In addition, carrier proteins may provide T-cell epitopes that recruit T-cell help for the humoral immune response.^32^ While we are currently testing EPA conjugate vaccines in clinical trials for safety and efficacy, we continue to evaluate other vaccine delivery platforms for more effective TBV candidates.

Membrane vesicles have garnered considerable interest as a vehicle for drug and vaccine delivery.^36^ The outer membrane vesicles (OMV) of gram-negative bacteria have been of particular interest for vaccines due to their particle structure and inherent immune modulatory properties.^37,38^ OMV from *Neisseria meningitidis* serogroup B, termed outer membrane protein complex (OMPC), is the platform for the polyribosylribitol phosphate (PRP) conjugate vaccine approved for *Haemophilus influenzae* type b (Hib) (PedvaxHIB®)^39–41^ and a component of meningococcal-B vaccine (Bexsero®),^42^ establishing OMVs as a safe and effective vaccine delivery platform. We evaluated OMPC as a carrier for Pfs25 and observed enhanced immune response compared to un-conjugated antigen,^43^ and a number of other antigens have been similarly assessed elsewhere.^44–47^ OMPC consists of several proteins embedded in the membrane that can serve as sites for chemical conjugation of antigens. By controlling synthetic parameters, the number of antigens conjugated on the OMPC surface can be modulated. Here, we describe the synthesis of a series of Pfs230-OMPC conjugates with varying levels of antigen load as well as their evaluation in mouse immunogenicity studies using alum formulations. Pfs230 used in this study is a 22 kDa recombinant protein corresponding to domain-1 (amino acids Ser^542^- Gly^736^) of Pfs230. Chemical conjugation of Pfs230 to OMPC resulted in significant enhancement in antibody response against Pfs230 as well as transmission blocking activity. Immunogenicity did not significantly vary within the range of antigen load tested, and OMPC conjugates showed a substantial dose-sparing effect compared to the EPA conjugate. In antibody subclass analysis, EPA conjugates induced IgG1-dominant immune responses whereas OMPC conjugate responses were dominated by IgG2.

## Results

### Conjugate synthesis

Pfs230-OMPC conjugates were synthesized by methods similar to those described earlier for chemical conjugation of Pfs25 to OMPC^43^ (Method 1; Supplementary Fig. 1). Pfs230 was reacted with N-(ε-Maleimidocaproyloxy) succinimide ester (EMCS) to generate the antigen with maleimide moieties. Desired number of modifications were achieved by varying the input ratio of antigen to EMCS. Separately, OMPC was reacted with N-acetylhomocysteine thiolactone to modify amino groups of membrane anchored proteins and generate free thiol groups on the OMPC surface. Levels of OMPC and Pfs230 modifications were determined by DTDP and reverse-DTDP assay, respectively.^33,48^ Maleimide-modified antigen and thiolated OMPC were mixed to allow chemical conjugation through thioether bond formation.

In an alternate method of chemical conjugation (Supplementary Fig. 1; Method 2), Pfs230 was reacted with N-Succinimidyl-S-acetylthioacetate (SATA) to generate protected thiol moieties on amino groups, yielding an average 3 protected thiols per antigen molecule. OMPC was reacted with EMCS to generate maleimide-modified OMPC. Prior to conjugation, SATA-derivatized antigen was treated with hydroxylamine to de-protect the thiol groups and combined with maleimide modified OMPC to generate the conjugate.

Conjugates with different antigen load (relative amount of antigen conjugated to OMPC) were prepared using method 1 by varying the level of modification and input ratio of modified antigen and OMPC in the conjugation step (Table 1). Antigen loads for each conjugate were determined by a non-linear regression analysis of amino acid analysis data obtained for antigen, OMPC and conjugate, a method routinely used on protein-protein conjugates in our laboratory.^49^ Antigen load for Pfs230 by weight varied from 6 to 17% of the total weight. Further increase in thiolation did not increase the antigen load, likely limited by steric restrictions. One conjugate, Pfs230-OMPC-5, was synthesized by method 2 and resulted in 12.5% antigen load.

**Table 1:** Characteristics and synthetic parameters of Pfs230-OMPC conjugates

Conjugate	Lot #	Conjugation Chemistry	# Maleimide or thiols per antigen	# thiols or maleimide per OMPC	Antigen input (mg)	OMPC input (mg)	Thiol/Maleimide (input ratio)	Antigen Load (wt.%)	Antigens/OMPC (#)
Pfs230-OMPC-1	8151	Method 1	0.28 (mal)	3640 (thiol)	7.62	7.03	6.7 (0.52)	6	109
Pfs230-OMPC-2	8132	Method 1	0.78 (mal)	1730 (thiol)	0.4	2.2	6.7 (0.15)	9.5	173
Pfs230-OMPC-3	8169	Method 1	2.55 (mal)	2200 (thiol)	10.1	21.4	1.0 (0.32)	12	218
Pfs230-OMPC-4	8167	Method 1	1.69 (mal)	3465 (thiol)	18.3	16.2	1.0 (0.53)	17	310
Pfs230-OMPC-5	8133	Method 2	2.8 (thiol)	736 (mal)	1.16	4.5	2.1 (0.20)	12.5	228

### Characterization of conjugates

OMPC conjugates were examined by SDS-PAGE and Western blot for evidence of antigen conjugation (Supplementary Fig. 2). On SDS-PAGE (Supplementary Fig. 2A), unmodified OMPC showed several bands representing multiple proteins present in OMPC and thiol modification did not alter their apparent molecular weights. After conjugation to antigen, most OMPC bands became more diffuse or smeared with a shift towards higher molecular weights. In western blot analysis using anti-Pfs230 antibody 4F12 (Supplementary Fig. 2B), Pfs230-OMPC conjugates synthesized by both method 1 and method 2 showed strong staining with 4F12 whereas modified and unmodified OMPC failed to stain, clearly demonstrating conjugation of Pfs230 to OMPC by the two different synthetic methods (Supplementary Fig. 2B).

### Conjugate composition

Composition of OMPC conjugates were determined by amino acid analysis. Amino acid compositions of Pfs230, OMPC, and conjugates were determined independently and a selected set of 13 amino acids common to all three was analyzed by nonlinear regression analysis with least square fitting to estimate the weight of antigen in the total weight of the conjugate, based on the method of Shuler.^49^ Using a molecular mass of 40,000,000 Daltons for OMPC,^47^ the average number of Pfs230 per OMPC was calculated, which ranged from 109 to 310 (Table 1).

### Structural integrity

**Fig. 1:**
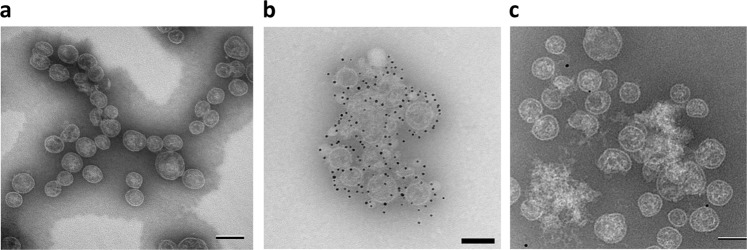
Electron microscopy of OMPC and Pfs230-OMPC conjugate. **a** Transmission electron microscopic image of OMPC. **b** Transmission electron microscopic image of Pfs230-OMPC-3 stored at 4 °C for one year, after incubation with anti-Pfs230 antibody, 4F12 (1:10), followed by gold-labeled secondary antibody. **c** Transmission electron microscopic image of Pfs230-OMPC-3 incubated with gold-labeled secondary alone (omitting incubation with primary antibody, 4F12). Images were taken on a FEI Biotwin Tecnai microscope and collected on an AMT XR611 camera system. Scale bar: 100 nm

The expected vesicular structure of OMPC was confirmed by transmission electron microscopy (TEM)^50^ and was not disrupted by the chemical conjugation to the antigen (Fig. 1a). Immuno-electron microscopic (IEM) using a primary antibody against Pfs230 (4F12) and a gold-conjugated secondary antibody with TEM imaging confirmed the presence of antigens on OMPC (Fig. 1b), whereas omission of 4F12 yielded no reactivity with secondary antibody alone (Fig. 1c). Gold labeling (black spots) clearly shows the presence and location of Pfs230 on OMPC, largely on the vesicle surface. These images also show that OMPC maintains its vesicular structure after conjugation.

### Formulation and stability of OMPC conjugate in AdjuPhos^®^

All OMPC conjugates were adsorbed on to AdjuPhos^®^ (0.45 mg/ml aluminum content) after synthesis to allow long term storage of the conjugates at 4 °C^45^. We evaluated the stability of the OMPC conjugate one year after the synthesis using IEM. Pfs230-OMPC conjugate stored at 4 °C adsorbed on AdjuPhos^®^ showed labeling of the Pfs230 antigen conjugated to OMPC in IEM studies (Fig. 1). In contrast, IEM image of the same conjugate stored in the absence of AdjuPhos^®^ (Supplementary Fig. 3) shows extensive labeling not associated with OMPC, indicating the dissociation or degradation of Pfs230 from OMPC. This confirms that adsorption of OMPC conjugates on AdjuPhos^®^ stabilizes the conjugate for long term storage at 4 °C. All experiments described here were carried out within one year of the synthesis.

### Immunogenicity of conjugates

**Fig. 2:**
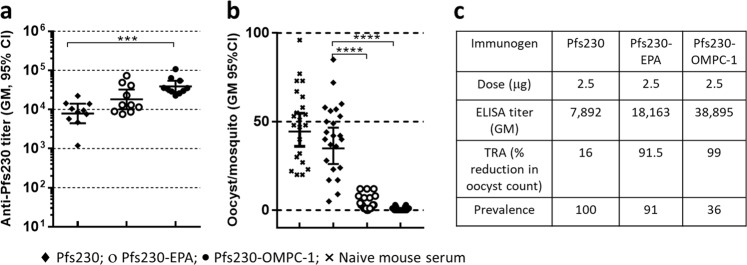
Effect of chemical conjugation on the immunogenicity of Pfs230. **a** Anti-Pfs230 antibody titer of Pfs230, Pfs230-EPA, and Pfs230-OMPC-1 in mice immunized twice (on days 0 and 28) with 2.5 µg antigen dose per mouse. ELISA was performed using sera obtained on day 42 using Pfs230 as plate antigen. **b** SMFA results showing the number of oocysts developed in each of the mosquitos fed on *P. falciparum* gametocytes mixed with immune sera obtained from mice immunized with Pfs230, Pfs230-EPA and Pfs230-OMPC-1 along with mosquitos fed on naïve mouse sera as control. SMFA was carried out using pooled sera for each group (10 mice in each group) after 1–5 dilution of sera. **c** Table listing the % reduction in oocyst count in mosquitos fed on immune sera compared to control sera for each group along with the antibody titer of the sera used as well as the prevalence data (% of mosquitos with at least one oocyst). Mice were immunized with conjugates formulated with AdjuPhos^®^. Error bars represent 95% confidence limit of the geometric mean. Statistical differences between groups were measured using a Kruskal–Wallis One-way ANOVA followed by a Dunn multiple comparator test. *p ≤ 0.05, **p ≤ 0.01, ***p ≤ 0.001, ****p ≤ 0.0001

We tested Pfs230-OMPC conjugates in mouse immunogenicity studies to evaluate antibody response and functional activity of immune sera. Unconjugated Pfs230 and a chemical conjugate of Pfs230 with EPA (Pfs230-EPA) were used as comparators in the mouse studies. CD-1 mice immunized with Pfs230, Pfs230-EPA and Pfs230-OMPC-1 (2.5 μg Pfs230 antigen each) formulated in 0.45 mg/ml AdjuPhos^®^ (aluminum content) on days 0 and 28 had sera collected on day 42 for analyses. While Pfs230 alone induced high antibody titer, chemical conjugation to EPA or OMPC further increased titers, with OMPC conjugate generating significantly higher titer compared to Pfs230 alone (Fig. 2a).

Functional activity of immune sera was evaluated in standard membrane feeding assay (SMFA) as its ability to inhibit the oocyst development in the mosquito midgut after a blood meal consisting of immune serum and cultured gametocytes. Activity was quantified as the reduction in the average oocyst count per mosquito [transmission-reducing activity (TRA) or percentage of mosquitos with at least one oocyst in the midgut (prevalence)]. Pooled sera for each group were used for SMFA analysis and naïve mouse serum was used as control. Sera from mice immunized with Pfs230 showed modest decrease (16%) in oocyst count compared to the control, indicating low level of functional activity. Sera from mice immunized with Pfs230-EPA and Pfs230-OMPC conjugates showed significantly greater reduction in oocyst count versus Pfs230 monomer, with TRA of 91.5 and 99%, respectively. OMPC conjugate also showed low infection prevalence indicating high transmission blocking activity by this conjugate (Fig. 2c).

### Dose response

**Fig. 3:**
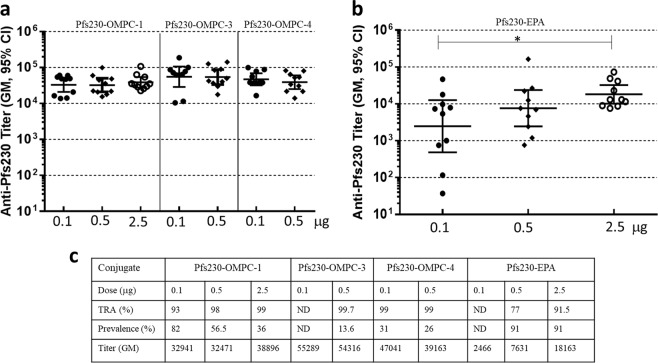
Effect of antigen dose on anti-Pfs230 antibody titer of mice immunized with Pfs230 conjugates of OMPC and EPA. **a** Anti-Pfs230 antibody titer observed in sera of mice vaccinated with 3 different Pfs230-OMPC conjugates and different doses. **b** Serum antibody titer observed in sera of mice vaccinated with 3 different doses of Pfs230-EPA conjugate. Mice were immunized on days 0 and 28 with conjugates at stated doses formulated in AdjuPhos^®^ and sera collected on day 42 were analyzed by ELISA. **c** Table showing the functional activity, obtained by SMFA, of OMPC and EPA conjugates at various doses in terms of TRA (% reduction in oocyst count compared to control) and prevalence (% of dissected mosquitos with at least one oocyst in the midgut). Table also shows the antibody titer of the sera, which were diluted 1 to 5-fold for SMFA. Error bars represent 95% confidence limit of the geometric mean. Statistical differences between groups were measured using a Kruskal–Wallis one-way ANOVA followed by a Dunn multiple comparator test. *p ≤ 0.05

We examined the effect of antigen dose on immunogenicity of Pfs230-OMPC conjugates in the dose range of 0.1–2.5 μg Pfs230 antigen/vaccination. Figure 3 compares the serum antibody titer (Fig. 3a) and functional activity (Fig. 3c) of three Pfs230-OMPC conjugates with Pfs230-EPA conjugate (Fig. 3b, c), on Day 42 after two vaccinations on days 0 and 28 for each dose. Pfs230-OMPC-1 induced high antibody titers that did not vary significantly in the 0.1–2.5 μg dose range, and similar results were seen with two other Pfs230-OMPC conjugates tested at 0.1 and 0.5 μg doses (Fig. 3a). In contrast, EPA conjugates showed a clear dose response with significant differences in titer between 0.1 and 2.5 μg doses (Fig. 3b). At the lowest dose (0.1 μg) antibody titer for all OMPC conjugates were significantly higher (*p* ≤ 0.0056) than EPA conjugate. At this dose, OMPC conjugates induced high functional activity (>90% TRA) (Fig. 3c); SMFA data were not obtained for EPA conjugate at this dose. Although OMPC conjugate titers at 0.5 and 2.5 μg doses were not significantly higher than EPA conjugates, their transmission reducing activity at these doses were significantly higher (*p* ≤ 0.0004 and *p* = 0.0227, respectively). Furthermore, Pfs230-OMPC-4 at 0.1 μg dose induced significantly greater TRA (*p* < 0.0001) than EPA conjugate at 2.5 μg dose, indicating a substantial dose sparing effect of OMPC conjugates. OMPC conjugates also gave consistently low mosquito infection prevalence suggesting high transmission blocking activity by OMPC conjugates (Fig. 3c).

### Effect of antigen load

We assessed the effect of antigen load on immunogenicity of OMPC conjugates using conjugates with antigen load ranging from 6 to 17% by weight (109–310 antigens per OMPC). Antigen dose per vaccination was 0.5 μg in terms of Pfs230 equivalent for all conjugates, and sera were collected 2 weeks after dose 2. We observed similar antibody titer for all 4 conjugates with no significant differences (Supplementary Fig. 4).

### Antibody subclass analysis

**Fig. 4:**
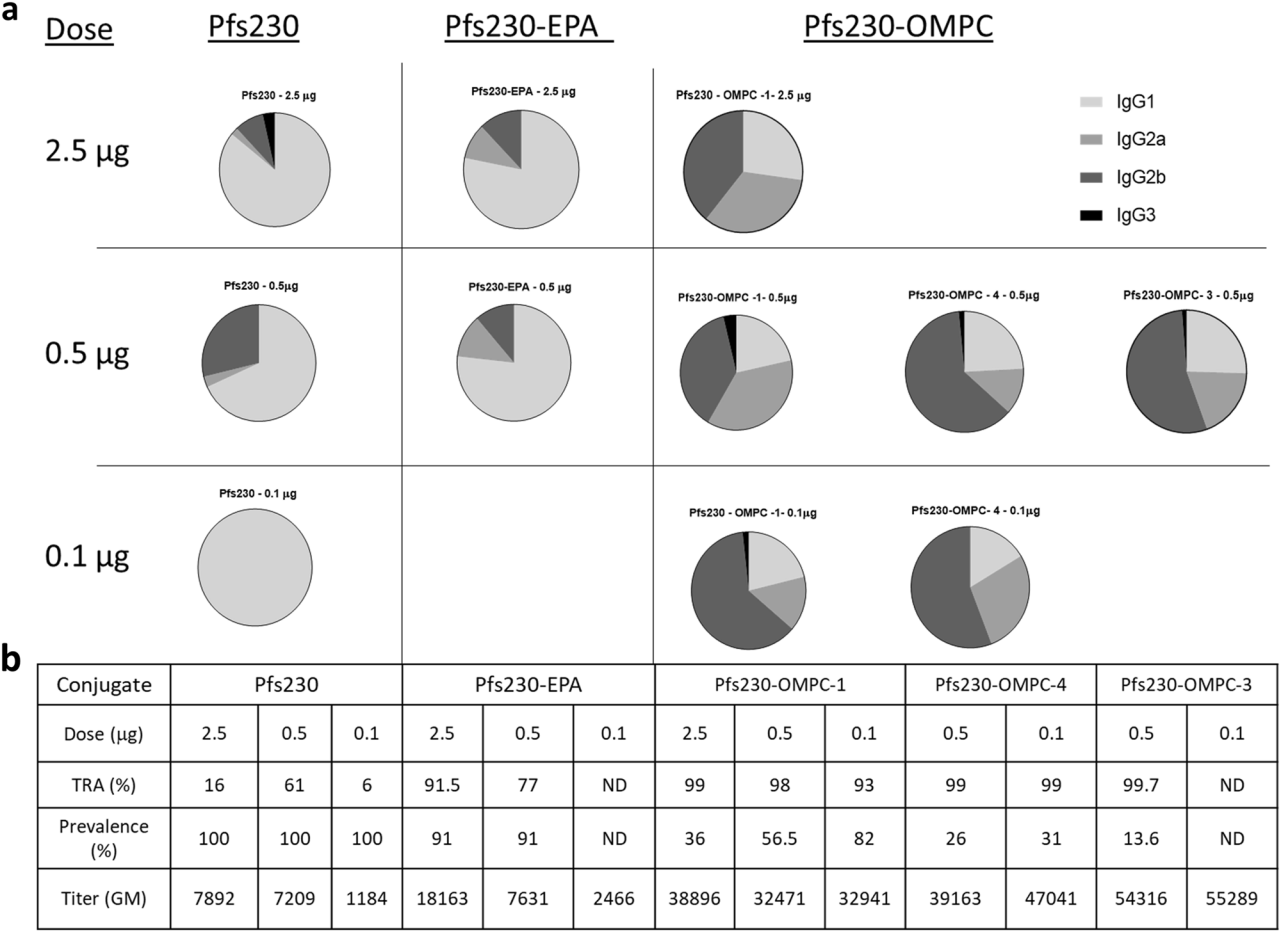
IgG subclass distribution in the immune sera. **a** IgG subclass (IgG1, IgG2a, IgG2b, and IgG3) distribution of sera from mice vaccinated with Pfs230, Pfs230-EPA and three different Pfs230-OMPC conjugates at different doses. Immune sera collected on day 42 after two vaccinations (days 0 and 28) were analyzed by ELISA. **b** Table listing the TRA, prevalence and antibody titer of sera analyzed for IgG subclass distribution. ND: not determined due to lack of enough serum samples

Pooled mouse sera from each group that received Pfs230 vaccine formulated in AdjuPhos® were analyzed for IgG1, IgG2a, IgG2b, and IgG3 distributions (Fig. 4). Pfs230 monomer and Pfs230-EPA conjugate generated an IgG1 dominant (≥70%) response, and Pfs230 monomer at lowest dose gave exclusively IgG1 response. In contrast, all three OMPC conjugates of Pfs230 showed an IgG2 dominated profile with ≥70% IgG2 (IgG2a + IgG2b), 16–27% IgG1 and in some cases a minor contribution from IgG3. This general profile was maintained at the different doses used, albeit the proportion of IgG2a and IgG2b varied with dose for one conjugate (Pfs230-OMPC-1).

### Alternate synthesis and duration of immune response

**Fig. 5:**
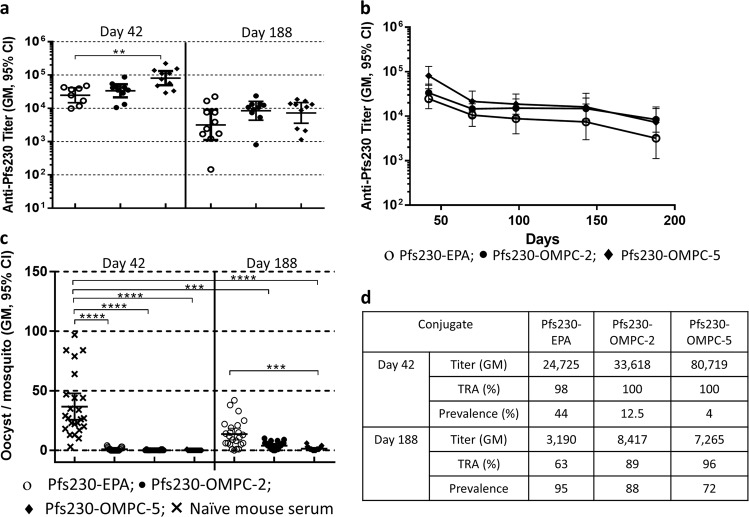
Immunogenicity of conjugates synthesized by the two different methods. **a** Comparison of serum anti-Pfs230 antibody titer of Pfs230-OMPC conjugates synthesized by method 1 (Pfs230-OMPC-2), method 2 (Pfs230-OMPC-5) and Pfs230-EPA assayed on day 42 and day 188. Mice were immunized on days 0 and 28 at 0.5 µg dose with conjugates formulated in AdjuPhos^®^. **b** Anti-Pfs230 antibody titer of Pfs230-OMPC-2, Pfs230-OMPC-5, and Pfs230-EPA at different time points up to day 188, assessing the durability of immune response. **c** Reduction in the midgut oocyst count of mosquitos fed on the immune sera from mice vaccinated with the above three conjugates on days 42 and 188, compared to mosquitos fed on control sera, assayed by SMFA for functional activity. **d** Table listing the functional activity (TRA and prevalence) of the three conjugates, along with their titer. Error bars represent 95% confidence limit of the geometric mean. Statistical differences between groups were measured using a Kruskal–Wallis one-way ANOVA followed by a Dunn multiple comparator test. *p ≤ 0.05, **p ≤ 0.01, ***p ≤ 0.001, ****p ≤ 0.0001

Synthetic method 1 requires generation of a substantial number of thiols on the OMPC surface. Thiolated OMPC must be rapidly reacted with maleimide functionalized antigen to avoid disulfide formation between adjacent thiols and loss of free thiol moieties on OMPC surface. To address this, an alternate synthetic procedure was developed where OMPC was modified with maleimide moieties, using EMCS reagent. Pfs230 was modified with approximately three protected thiols using SATA reagent. Both EMCS-modified OMPC and SATA-modified Pfs230 can be stored for longer period and reduces the need for immediate conjugation. Protected thiols on Pfs230 were deprotected prior to conjugation with maleimide modified OMPC (method 2). One conjugate, Pfs230-OMPC-5, was synthesized using this method and its immunogenicity and functional activity were compared to a conjugate made by method 1 (Pfs230-OMPC-2) and Pfs230-EPA. Though Pfs230-OMPC-5 titer was not significantly higher than Pfs230-OMPC-2, (Fig. 5a, left panel), it gave a significantly higher titer than EPA conjugate (Fig. 5a, left panel). Mice immunized with these three conjugates were monitored for a longer period of time to evaluate the durability of their immune response. All three conjugates maintained significant antibody titers during the time course until day 188 (Fig. 5a, b, right panel). Antibody levels decreased similarly for all the conjugates during the time course while OMPC conjugates maintained higher titers than EPA during the period.

Functional activity of the conjugates was assessed on days 42 and 188 (Fig. 5c, d) by SMFA. On day 42, all three conjugates induced high functional activity with TRA ≥98%, significantly greater than control sera. On day 188, TRA of the EPA conjugate was reduced to 63% while two OMPC conjugates retained high functional activity (89 and 96% TRA for Pfs230-OMPC-2 and Pfs230-OMPC-5, respectively). On day 188, while both OMPC conjugates showed significantly higher functional activity compared to control sera, Pfs230-OMPC-5 showed a significantly higher functional activity compared to EPA conjugate.

## Discussion

Pfs230 is a large protein expressed on the gametocyte and gamete surface, and immunization with this antigen induces potent transmission blocking antibodies. Expression of full-length Pfs230 has not been achieved and smaller domains have been evaluated as candidate vaccines. We therefore used a recombinant protein of domain-1 with molecular weight of 22 kDa, expressed in *Pichia pastoris*, as our candidate antigen.^22^ Immunogenicity and functional activity of this antigen can be enhanced by its conjugation to a carrier protein, EPA (Fig. 2) and an EPA conjugate of this antigen is currently under clinical evaluation.^51,52^ In this study, we examined OMPC as an alternate carrier for Pfs230, evaluated its immunological responses, and compared it to a Pfs230-EPA conjugate in alum formulations.

OMPC as an antigen delivery platform offers several advantages. Since OMPC is a carrier in licensed vaccines, its safety and efficacy are well-established.^53,54^ Chemical conjugation to OMPC enhances immunogenicity of polysaccharide antigen.^55^ OMPC provides a particle platform for antigen display and possesses inherent adjuvant activity.^38,39,56–58^ In a previous study, we demonstrated that chemical conjugation of Pfs25 (another transmission blocking antigen) to OMPC enhances immunogenicity against Pfs25 in mouse and NHP studies.^43^ OMPC provides several conjugation sites on its surface due to presence of several embedded proteins in its membrane.

To evaluate OMPC as a carrier for Pfs230, we synthesized a series of conjugates with varying number of antigens on the OMPC surface using thioether chemistry that is also employed for the synthesis of the Pfs230-EPA conjugate vaccine candidate currently under clinical evaluation. All OMPC conjugates were synthesized and purified under sterile conditions and conjugates were adsorbed on to AdjuPhos^®^ immediately after synthesis. Of the 5 conjugates evaluated in mouse studies, 4 were synthesized by method 1 and one was synthesized by method 2 (Supplementary Fig. 1). In method 1, OMPC surface was modified with large number of thiol (−SH) groups that were used for conjugation with maleimide modified Pfs230. This method requires immediate reaction with maleimide-modified antigen to avoid loss of reactive thiols due to intramolecular disulfide formation. In the alternative method (method 2), OMPC surface was modified with stable maleimide moieties and Pfs230 was modified with protected thiol moieties which enabled its storage until deprotection and coupling to modified OMPC. With this approach, both OMPC and antigen modifications remain stable, reducing the need for immediate reaction. This method also offers better control of each step and characterization of modified intermediates and may be more suitable for manufacturing and scaleup.

Mouse immunogenicity studies comparing OMPC conjugate of Pfs230 with its EPA conjugate and Pfs230 alone in alum adjuvant showed that chemical conjugation of Pfs230 to OMPC or EPA increased the antibody titer against Pfs230. Both conjugates gave high transmission reducing activity in standard membrane feeding assay. Antibody titer and transmission reducing activity of immune sera obtained for OMPC conjugate were significantly greater than un-conjugated Pfs230 (Fig. 2). Immunogenicity of OMPC conjugates with different antigen load indicate that for a given antigen dose, antigen load of the conjugate does not significantly affect the immune response (Supplementary Fig. 4). In contrast to EPA conjugate, dose response of OMPC conjugates in 0.1–2.5 µg dose range did not show any significant dose-dependent differences in antibody titer against Pfs230 (Fig. 3a). OMPC conjugates generally yielded higher antibody titer than EPA conjugates with greater differences at lower doses. At 0.1 µg dose, OMPC conjugates gave 13-fold to 22-fold higher antibody titer; at 0.5 µg dose, the difference was 4-fold to 7-fold. Functional activity of all three OMPC conjugates also were high (>93%) at different doses while EPA conjugate showed a decrease with decreasing dose. This demonstrates a substantial dose sparing effect of OMPC conjugates in comparison to EPA conjugate of Pfs230 and further studies are necessary to determine the minimum effective dose for the OMPC conjugate. In addition, mice immunized with OMPC conjugates retained high antibody titer and functional activity for a longer period. OMPC conjugate synthesized by method 2 showed significantly higher functional activity on day 188 compared to EPA conjugate (Fig. 5) indicating that this more convenient method of synthesis generates equally immunogenic or superior conjugates. This needs to be confirmed further by evaluating additional conjugates synthesized by method 2.

OMPC is expected to enhance the immunogenicity of a conjugated antigen by two different mechanisms. It presents the conjugated antigen in a particulate structure and provides adjuvant activity that enhances the immunogenicity of the conjugated antigen.^38,56–58^ OMPC has been shown to possess TLR2-mediated adjuvant activity attributed to its membrane porin proteins.^57^ Furthermore, since OMPC is derived from the outer membrane of gram-negative bacteria, it may also contain residual TLR4 ligands such as lipopolysaccharides.^38,56–58^ TLR4 adjuvants are known to induce a Th1-biased immune response whereas TLR2 skews the response in Th2 direction.^59^ Additionally, OMPC conjugates were adsorbed on AdjuPhos^®^, which also is a Th2-biased adjuvant. Our results indicate a Th1-dominant immune response for OMPC conjugates, with high levels of IgG2 induction in immunized mice (Fig. 4). IgG2 is more efficient in complement activation,^60^ and therefore may contribute to the high functional activity of Pfs230-OMPC conjugates.^61–63^ Additional studies, including evaluation of cellular responses, are required to delineate how different adjuvants may impact the functional activity induced by OMPC or EPA conjugates.

In this initial study, we evaluated the feasibility of using OMPC as a delivery platform for Pfs230 antigen. For this conjugate to be a viable malaria vaccine candidate, it should be amenable to large scale manufacturing under cGMP at a reasonable cost. OMPC is a delivery platform for a polysaccharide conjugate vaccine currently approved for use (PedvaxHIB®). Therefore, the technology for development of a vaccine based on OMPC conjugate at a reasonable cost already exists. Thioether chemistry used for conjugation of Pfs230 to OMPC is also used for synthesis of Pfs230-EPA and involves the same number of synthesis and purification steps. Pfs230-EPA is currently manufactured under cGMP and is undergoing large scale clinical trials. Therefore, it is feasible to manufacture Pfs230-OMPC conjugate at a larger scale under cGMP using currently available technologies. In mouse immunization studies described here, Pfs230-OMPC appears to be a more potent conjugate compared to Pfs230-EPA. This may be attributable to the induction of an immune response more appropriate to the functional activity of Pfs230 vaccine. Whether this holds true in higher animals and in humans is yet to be determined.

In conclusion, OMPC is a promising carrier for the TBV antigen, Pfs230. Chemical conjugation of Pfs230 to OMPC significantly increased the immunogenicity and transmission blocking activity of the conjugated antigen. OMPC conjugates of Pfs230 showed superior antibody response and functional activity compared to EPA conjugates, especially at lower doses. The Th1-biased antibody profile induced by OMPC conjugates is well suited to augment the complement-dependent biological function of a Pfs230 vaccine.

## Methods

All syntheses and processing of OMPC conjugates and their intermediates were performed in a biosafety cabinet or under sterile conditions. Sterile laboratory supplies were used for synthesis and processing steps, and all buffers used were sterilized by sterile filtration. OMPC conjugates were adsorbed on AdjuPhos^®^ (0.45 mg/ml aluminum content) immediately after synthesis for long term storage at 4 °C.

### Synthesis of Pfs230-OMPC conjugates—Method 1

#### Buffers

Buffer A = 25 mM HEPES + 154 mM NaCl pH7.3; Buffer B = 25 mM MES pH6.1; Buffer C = 0.11 M Sodium Tetraborate pH 11.3; Buffer D = Nitrogen-sparged Milli-Q-water; Buffer E = 10 mM HEPES + 154 mM NaCl pH 7.3; EDTA/DTT solution = 100 mM EDTA + 35.3 mM DTT in Buffer C; Quenching Solution = 100 mM N-Ethylmaleimide; TED Buffer = 100 mM Tris + 10 mM EDTA + 0.5% Deoxycholate pH 8.5.

#### Maleimide-modified Pfs230 (Pfs230-mal)

To a 2.451 ml solution of 1.508 mg (6.902 × 10^−8^ moles) of Pfs230 in Buffer A was added 25.1 μl (118 μg, 2.70 × 10^−7^ moles) of a 4.69 mg/ml solution of Sulfo-SMCC in Buffer A. The final protein concentration was 0.609 mg/ml. This reaction mixture was briefly vortexed and placed in the dark overnight at 4 °C. The reaction mixture was then concentrated by centrifugal filtration using a 4-ml CFD3 for 15 min at 5 °C. The ~0.8 ml retentate was desalted across a PD10 column which had been pre-equilibrated with Buffer B. The initial sample was chased with 1.7 ml of Buffer B, and Pfs230-mal was eluted by 2.0 ml Buffer B. An indirect DTDP assay was used to determine the maleimide concentration which was 11.9 µM. From the protein concentration, average molar ratio of maleimides to Pfs230 was estimated to be 0.78 and the yield was 0.669 mg of Pfs230-mal.

#### Thiol-modified OMPC (OMPC-SH)

To a 900 µl solution of 6.28 mg (1.57 × 10^−10^ moles) of OMPC in Buffer C was added 150 μl of EDTA/DTT solution and 75 µl (29.9 mg, 1.88 × 10^−4^ moles) of a 91.5 mg/ml solution of NAHT in Buffer D. The final OMPC protein concentration was 5.59 mg/ml. This reaction mixture was left to incubate at +4 °C for 2 h in the dark. The OMPC-SH was then pelleted by ultracentrifugation (TLS55 rotor in Optima MAX Benchtop ultracentrifuge, 197,000 × *g*, 4 °C, 45 min). Pellet was resuspended in 0.7 ml of Buffer D using a Dounce homogenizer. The homogenate was pelleted again by ultracentrifugation. Pellet was resuspended in 0.7 ml Buffer B using a Dounce homogenizer and recovered. Assay of thiol moieties by direct DTDP assay determined the thiol to OMPC molar ratio to be 1730.

#### Pfs230-OMPC-2 conjugate

To a 1.20 ml solution in an amber-colored microcentrifuge tube containing 402 µg (1.84 × 10^−8^ moles) of Pfs230D1M-mal in Buffer B was added 246 µl of OMPC-SH in Buffer B. The reaction mixture had a molar ratio of 6.6 thiols per maleimide. The conjugation reaction mixture was placed at 4 °C in the dark. After 16 h, 4.76 µl of Quenching Solution was added to the conjugation reaction, equaling a five-molar excess of maleimides (NEM) per thiol, and the reaction was left at room temperature for three more hours. The 1.451 ml of quenched reaction was then transferred into a 5 ml Float-A-Lyzer dialysis device with a MWCO of 100 kDa and dialyzed for 4 h at room temperature against 500 ml of TED Buffer. The sample was then dialyzed five times against 350-fold excess volume of Buffer E at 4 °C with about 4 h between buffer changes. After the final dialysis, the retentate was centrifuged for 5 min at 1000 RCF at 5 °C, and 3.00 ml of supernatant was recovered as the final bulk conjugate. A modified SDS-Lowry assay^64^ determined the protein concentration to be 0.889 mg/ml. Amino acid analysis of the conjugate showed that Pfs230 is 9.5% (w/w) of total protein mass of the conjugate. The Pfs230 concentration of the bulk conjugate was calculated to be 84.5 µg/ml, and the yield of Pfs230 conjugated to OMPC was calculated to be 254 µg. Percentage recovery of Pfs230 from the conjugation and processing steps was calculated to be 63% and for the overall process to be 28%.

Pfs230-OMPC conjugates with different antigen loads were synthesized by identical procedure as described above except for the difference in the input of various reagents, as listed in Table 1.

### Synthesis of Pfs230-OMPC conjugates—Method 2

#### Buffers

Buffer A: pH 7.2 PBSE (100 mM sodium phosphate, 150 mM NaCl, 5 mM EDTA) (prepared from BupH PBS Pack, Pierce # 28372); Buffer B: pH 6.5 PBSE (100 mM sodium phosphate, 150 mM NaCl, 5 mM EDTA) (prepared from BupH PBS Pack, Pierce # 28372); Buffer C: pH8.5 TED buffer (0.1 M Tris-HCl, 10 mM EDTA, 0.5% Sodium deoxycholate); Buffer D: pH 7.3 HEPES (10 mM HEPES, 0.15 M NaCl); Deacetylation Buffer: 0.5 M NH_2_OH in pH 7.2 PBSE.

#### Maleimide-activated OMPC (OMPC-mal)

A 1.0 ml suspension of OMPC (6.276 mg, 1.57 × 10^−10^ mol) in water was transferred to a 1 ml ultracentrifuge tube (Beckman #343778) and centrifuged at 197,000 × *g* (TLS55 rotor in Optima MAX Benchtop ultracentrifuge) at 4 °C for 45 min. The precipitate was re-suspended in 2 ml Buffer A (PBSE pH 7.2) and transferred to a 15 ml polypropylene round bottom tube. To this suspension 3.93 µl of a 30.8 mg/ml solution of EMCS in DMSO (0.121 mg, 3.92 × 10^−7^ mol) was added at once and the mixture was rotated for 90 min at room temperature. The resulting suspension was transferred into two 1 ml ultracentrifuge tubes and centrifuged at 197,000×*g* at 4 °C for 44 min. The precipitate was re-suspended in 1 ml sterile water, homogenized briefly by the dunce homogenizer and centrifuged again at 197,000 × *g* for 45 min. The precipitate was re-suspended in 0.5 ml buffer B (PBSE pH 6.5), transferred to a 2 ml dunce homogenizer and homogenized. Homogenized suspension was transferred to a 15 ml sterile polypropylene round bottom tube. 0.5 ml buffer B was used to rinse the homogenizer, combined with 0.5 ml homogenized sample to total 1 ml suspension. The protein concentration was estimated to be 5 mg/ml and maleimide modification was 736 maleimide per OMPC.

#### Thiol modified Pfs230 (Pfs230 -SH)

To a solution of 1.74 mg (7.97 × 10^−8^ mol) of Pfs230 in 0.87 ml of Buffer A at room temperature, 8.0 µl (0.184 mg, 7.97 × 10^−7^ mol) of a 23 mg/ml solution of SATA in DMSO was added at once with stirring. The resulting solution was stirred for 1 h at room temperature. The mixture was diluted with Buffer A and concentrated repeatedly to affect a 1000-fold buffer exchange using a CFD10 to give a final volume of approximately 0.7 ml (Concentration: 2 mg/ml).

To the resulting solution (0.7 ml) was added 0.07 ml of deacetylation buffer. The mixture was placed on a rotating shaker at 600 RPM for 1 h at room temperature. The mixture was transferred to a CFD10 and exchanged to Buffer B by repeated centrifugation and dilution to obtain a 1000-fold buffer exchange, yielding 0.34 ml of a 4.30 mg/ml solution of SH-modified Pfs230 (1.46 mg). The product was kept frozen at −80 °C until used. DTDP assay showed average 2.77 thiols per molecule of Pfs230. The yield of Pfs230-SH was 1.46 mg (83.9%).

#### Pfs230M-OMPC-5 conjugation

To 0.9 ml of a 5.0 mg/ml suspension of OMPC-mal in Buffer B (4.5 mg, 1.13 × 10^−10^ moles) 0.27 ml of a 4.30 mg/ml solution of Pfs230-SH (1.16 mg, 6.20 × 10^−8^ mol) in Buffer B was added, and the mixture was rotated for overnight at 4 °C. The excess maleimide groups were quenched by adding 18.2 µl of a 0.8 mg/ml solution of cysteine hydrochloride (0.015 mg, 8.31 × 10^−8^ mol) in Buffer B, and the mixture was rotated for an additional 15 min. The resulting solution was transferred to a 1 ml pre-wet float-A-Lyzer (100 K). It was dialyzed against 800 ml of Buffer C (TED buffer) for 4 h. Dialysis buffer was changed to 1000 ml Buffer D (HEPES pH 7.3) for 4 h. Dialysis was repeated for additional 4 times using Buffer D. Conjugate (1.3 ml) was recovered after the dialysis. Total protein recovery was 0.84 mg. Amino acid analysis showed Pfs230 to be 12.5% (w/w) of total protein which translate to 228 Pfs230 molecules per OMPC.

#### Analysis of conjugates and intermediates

Protein concentration of OMPC and OMPC conjugates were determined by modified Lowry protein assay^64^ according to manufacturer’s suggested protocol. Extent of thiol and maleimide modification of Pfs230 and OMPC were assayed by 4,4′-dithiodipyridine (DTDP) assay and reverse DTDP assay respectively.^33^ Composition of Pfs230-OMPC conjugates was analyzed by the method of Shuler, using amino acid analysis data.^49^ This method is used routinely in our laboratory for analyzing the composition of protein-protein conjugates.^33^ To determine the composition and antigen load of Pfs230-OMPC conjugates, amino acid analysis data of conjugate, antigen and OMPC were subjected to regression analysis with least square fitting, using a set of amino acids common to Pfs230 and OMPC. Antigen loads obtained for various conjugates are listed in Table 1.

#### Immunoelectron microscopy

Five microliter droplets of OMPC or Pfs230-OMPC conjugate in suspension were applied to 200 mesh freshly glow discharged carbon coated nickel grids and allowed to settle for 5 min. Excess was removed and then grids were incubated on 20 µl of Tris buffer with 1% tween 20, 0.1% BSA and 3% gelatin (blocking buffer) for 10 min. The grids were transferred to 10 µl droplets of primary anti-Pfs230 antibody (4F12) diluted 1:10 in blocking buffer and incubated for 45 min. After 3 × 5-min washes in blocking buffer, grids were incubated with 5 nm BBI colloidal gold goat anti-mouse IgG (H + L) (AH) (Ted Pella, Redding, CA) diluted 1:20 in blocking buffer for 45 min. After 1 × 5-min blocking buffer and 3 × 5-min dH_2_O washes, the grids were stained with NanoVan (Nanoprobes, Inc, Yaphank, NY) for 2 min and allowed to dry prior to viewing at 120 kV on a FEI Biotwin Tecnai microscope (Thermo Fisher Scientific, Hillsboro, OR). Digital images were collected on an AMT XR611 camera system (Advanced Microscopy Techniques, Woburn, MA) Scale bar: 100 nm

#### Immunogenicity studies

All animal studies were carried out per the guidelines and approval of the Institutional Animal Care and Use Committee (IACUC) at the National Institutes of Health. Immunogenicity of various Pfs230-OMPC conjugates and controls were evaluated in CD-1 mice (Charles River Laboratories). Groups of 10 mice were used for each test sample. Mice were vaccinated by intra-muscular injection of 50 µl formulations on Days 0 and 28. Blood samples from animals were collected by cardiac puncture, after anesthesia on day 42. Sera obtained were analyzed for anti-Pfs230 antibody titer by ELISA and functional activity by Standard Membrane Feeding Assay (SMFA).

#### ELISA and SMFA

Anti-Pfs230 antibody titers were assayed using standard ELISA method, with Pfs230 as the plate antigen. ELISA units were determined from the absorbance relative to reference antisera against Pfs230. Functional activity of immune sera was assayed using Standard Membrane Feeding Assay (SMFA) used for determining the transmission blocking activity.^29^ A set of *Anopheles* mosquitos (20–25) were fed on test sera mixed with cultured P. falciparum gametocytes, through a membrane feeding apparatus. After a week delay for development, each mosquito was dissected, and the number of oocysts developed in the midgut was counted. A reduction in the number of oocysts in the mosquito midgut fed on the immune sera compared to mosquitos fed on control sera indicates interruption of parasite development in mosquito midgut.

#### Statistical analysis

ELISA data were analyzed with Prism software (GraphPad Software, Inc., La Jolla, CA) and statistical differences between groups (*p* ≤ 0.05) were measured using a Kruskal–Wallis analysis followed by a Dunn multiple comparator test.

#### Antigens and carriers

Pfs230 (domain-1 of full length Pfs230; amino acids Ser^542^- Gly^736^; mol. wt. 21,854) was based on *P. falciparum* 3D7 allele sequence and were codon optimized and produced in *P. pastoris*, as described earlier.^22^ A suspension of OMPC in water was obtained from Merck & Co., Inc., Kenilworth, NJ, USA and had a protein concentration of 6.276 mg/ml. The carrier protein recombinant Exoprotein A (EPA) of *Pseudomonas aeruginosa* (molecular weight, 66,983 Da) was expressed in *E. coli*.

#### Pfs230-EPA

Pfs230-EPA is a chemically crosslinked conjugate of Pfs230 with a carrier protein, EPA. This conjugate, currently in clinical studies was used as a control in the evaluation of OMPC conjugates of Pfs230. Detailed synthesis and characterization of Pfs230-EPA conjugate by thioether chemistry is described in Scaria PV et al.^33^ Briefly, Pfs230 was modified with SATA to obtain approximately 3 protected thiol moieties per antigen. Carrier protein, EPA, was modified with EMCS to introduce about 11 molecules of maleimide per EPA. Modified antigen and carrier were characterized prior to conjugation. Prior to the synthesis of the conjugate, SATA modified Pfs230 was deprotected by addition of hydroxylamine to generate free sulfhydryl groups for reaction with maleimide. Sulfhydryl modified Pfs230 was then combined with maleimide modified EPA at 1:1 thiol to maleimide ratio to generate Pfs230-EPA conjugate. Composition of Pfs230-EPA conjugate used in this study, by amino acid analysis, showed that 54.6% (w/w) of the total protein in the conjugate is from Pfs230 antigen.

#### Chemicals and reagents

N-Ethyl maleimide (NEM), dl-*N*-Acetylhomocysteine thiolactone (“NAHT”) and N-Hydroxysulfosuccinimide (sNHS) were obtained from Sigma-Aldrich (St. Louis, MO). N-(ε-maleimidocaproyloxy)succinimide (EMCS), N-(ε-maleimidocaproyloxy) sulfo-succinimide sodium salt (sulfo-EMCS), Sulfosuccinimidyl 4-[N-maleimidomethyl] cyclohexane-1-carboxylate (“Sulfo-SMCC”) and S-acetylthioglycolic acid N-hydroxysuccinimidyl ester (SATA) were purchased from Pierce Biotechnology Inc. (Rockford, IL). CFD10 and CFD100 Amicon centrifugal filtration devices with 10 kDa and 100 kDa MWCO, respectively were obtained from Millipore (Billerica, MA).

### Reporting summary

Further information on experimental design is available in the Nature Research Reporting Summary linked to this article.

## Supplementary information

Supplementary Material with Legends

Reporting Summary

## Data Availability

The datasets generated during and/or analyzed during the current study are available from the corresponding author on reasonable request.

## Supplementary information

**Supplementary Information** accompanies the paper on the *npj Vaccines* website (10.1038/s41541-019-0121-9).
