# Supplementary material for: Outer membrane protein complex as a carrier for malaria transmission blocking antigen Pfs230
Source: NPJ Vaccines. 2019 Jul 8;4:24. doi: 10.1038/s41541-019-0121-9 (PMC6614402; doi:10.1038/s41541-019-0121-9)
Supplement: Supplementary file 1 — Supplementary Material with Legends [file 41541_2019_121_MOESM1_ESM.pdf]

Supplementary Figure 1. Schematics of Pfs230-OMPC conjugate syntheses by Method 1 and Method 2.

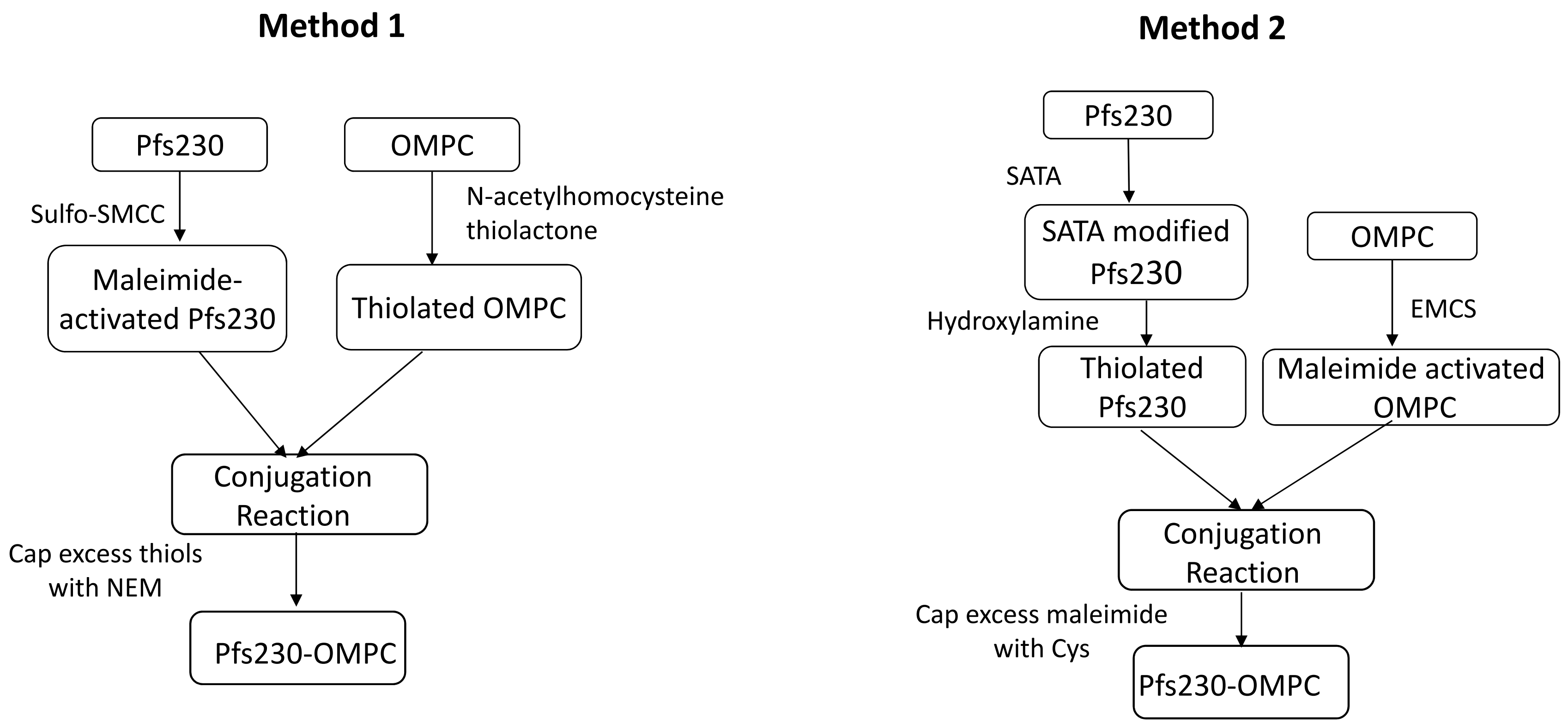

Supplementary Figure 2. SDS-PAGE and Western Blot analysis of Pfs230-OMPC conjugates synthesized by two different methods.

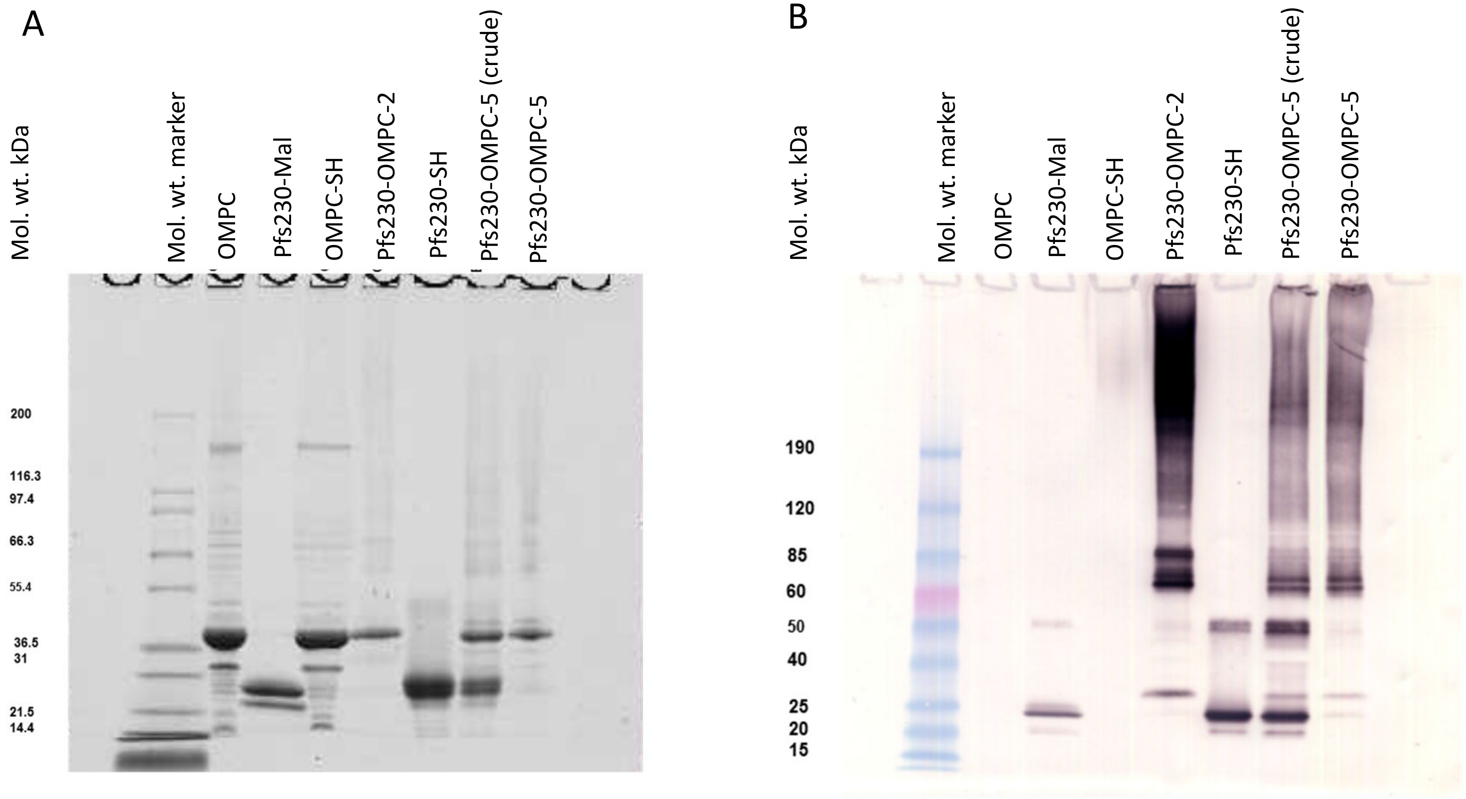

Supplementary Figure 3. Immuno-Electron Microscopy of unformulated Pfs230-OMPC conjugate stored at 4 °C.

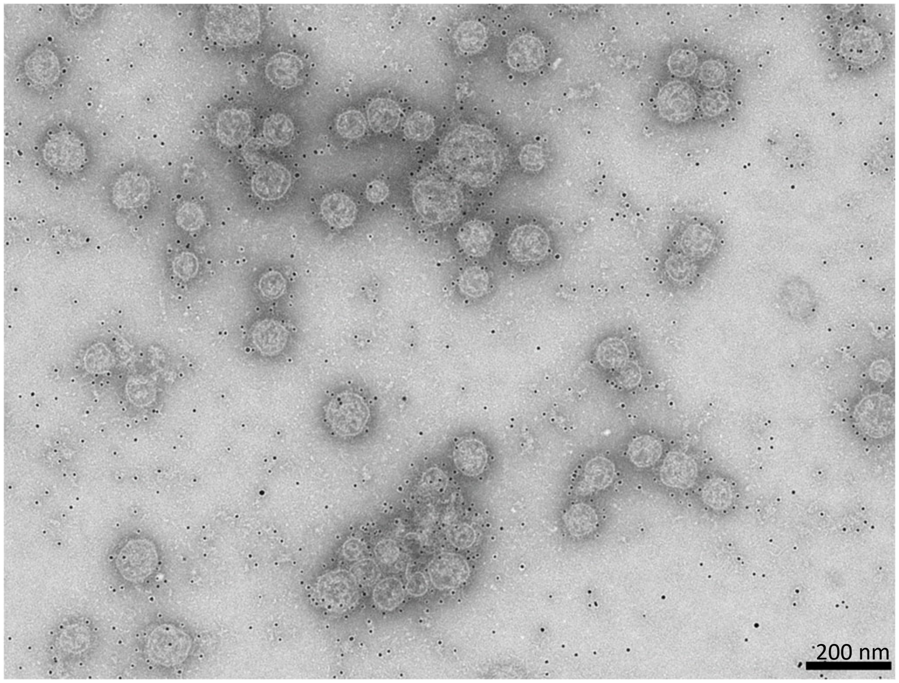

Supplementary Figure 4. Anti-Pfs230 antibody titer of Pfs230-OMPC conjugates with different antigen loads.

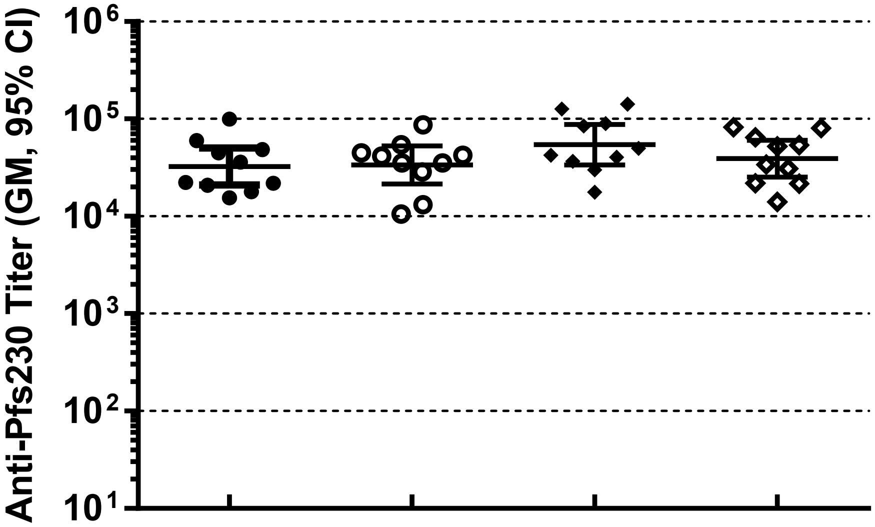

● Pfs230-OMPC-1; ο Pfs230-OMPC-2; ♦ Pfs230-OMPC-3; ◊Pfs230-OMPC-4

Supplementary Figures Legends:

Supplementary Figure 1. Schematics of Pfs230-OMPC conjugate syntheses by Method 1 and Method 2.

Supplementary Figure 2. SDS-PAGE and Western Blot analysis of Pfs230-OMPC conjugates synthesized by two different methods. (A) SDS-PAGE of OMPC, modified OMPC, Pfs230, modified Pfs230 and two OMPC conjugates, Pfs230-OMPC-2 (method 1) and Pfs230-OMPC-5 (method 2). (B) Western blot of the above gel developed by staining with anti-Pfs230 monoclonal antibody, 4F12 (1 µg/ml). Non-reducing SDS-PAGE was carried out with 4-12% gel and run in Tris-Glycine buffer. All blots derive from the same experiment and were processed in parallel.

Supplementary Figure 3. Immuno-Electron Microscopy of unformulated Pfs230-OMPC conjugate stored at 4 °C.

Transmission electron microscopic image of Pfs230-OMPC-3 after incubation with anti-Pfs230 antibody, 4F12 (1:10), followed by a gold-labeled secondary antibody. Conjugate was stored in the absence of AdjuPhos® at 4°C for one year after synthesis. Images were taken on a FEI Biotwin Tecnai microscope and collected on an AMT XR611 camera system. Scale bar: 200 nm.

Supplementary Figure 4. Anti-Pfs230 antibody titer induced by Pfs230-OMPC conjugates with different antigen loads. Groups of 10 CD-1 mice were vaccinated by intramuscular injection of 0.5 µg antigen (in terms of Pfs230) in 50 µl volume of various conjugates formulated in AdjuPhos® on days 0 and 28. Antigen load of conjugates varied from 6-17% (wt%). Sera obtained on day 42 were analyzed by ELISA using Pfs230 as coating antigen. ELISA data were analyzed

with Prism software (GraphPad Software, Inc., La Jolla, CA) and statistical differences between groups were measured using a Kruskal-Wallis One-way ANOVA followed by a Dunn multiple comparator test. Error bars represent 95% confidence limit of the geometric mean. * $p \leq 0.05$, ** $p \leq 0.01$, ***$p \leq 0.001$, **** $p \leq 0.0001$
